# Preoperative Texture Analysis Using ^11^C-Methionine Positron Emission Tomography Predicts Survival after Surgery for Glioma

**DOI:** 10.3390/diagnostics11020189

**Published:** 2021-01-28

**Authors:** Osamu Manabe, Shigeru Yamaguchi, Kenji Hirata, Kentaro Kobayashi, Hiroyuki Kobayashi, Shunsuke Terasaka, Takuya Toyonaga, Keiichi Magota, Yuji Kuge, Nagara Tamaki, Tohru Shiga, Kohsuke Kudo

**Affiliations:** 1 Department of Radiology, Saitama Medical Center, Jichi Medical University, 1-847, Amanuma-cho, Saitama-shi 330-8503, Saitama-ken, Japan; omanabe@jichi.ac.jp; 2 Department of Neurosurgery, Hokkaido University Graduate School of Medicine, Sapporo 060-8638, Japan; syamama1945@gmail.com; 3 Department of Diagnostic Imaging, Hokkaido University Graduate School of Medicine, Sapporo 060-8638, Japan; k.kobayashi@med.hokudai.ac.jp (K.K.); tshiga@med.hokudai.ac.jp (T.S.); kkudo@huhp.hokudai.ac.jp (K.K.); 4 Department of Neurosurgery, Kashiwaba Neurosurgery Hospital, Sapporo 062-8513, Japan; hiro-ko@med.hokudai.ac.jp (H.K.); terasas0727@gmail.com (S.T.); 5 PET Center, Department of Radiology and Biomedical Imaging, Yale University School of Medicine, New Haven, CT 06520, USA; takuyatoyonaga@gmail.com; 6 Division of Medical Imaging and Technology, Hokkaido University Hospital, Sapporo 060-8648, Japan; magota@huhp.hokudai.ac.jp; 7 Central Institute of Isotope Science, Hokkaido University, Sapporo 060-8638, Japan; kuge@ric.hokudai.ac.jp; 8 Department of Radiology, Kyoto Prefectural University, Kyoto 606-8522, Japan; natamaki@koto.kpu-m.ac.jp; 9 Global Station for Quantum Medical Science and Engineering, Global Institution for Collaborative Research and Education, Hokkaido University, Sapporo 060-8638, Japan

**Keywords:** methionine, positron emission tomography, glioma, texture analysis, prognosis

## Abstract

Background: Positron emission tomography with ^11^C-methionine (MET) is well established in the diagnostic work-up of malignant brain tumors. Texture analysis is a novel technique for extracting information regarding relationships among surrounding voxels, in order to quantify their inhomogeneity. This study evaluated whether the texture analysis of MET uptake has prognostic value for patients with glioma. Methods: We retrospectively analyzed adults with glioma who had undergone preoperative metabolic imaging at a single center. Tumors were delineated using a threshold of 1.3-fold of the mean standardized uptake value for the contralateral cortex, and then processed to calculate the texture features in glioma. Results: The study included 42 patients (median age: 56 years). The World Health Organization classifications were grade II (7 patients), grade III (17 patients), and grade IV (18 patients). Sixteen (16.1%) all-cause deaths were recorded during the median follow-up of 18.8 months. The univariate analyses revealed that overall survival (OS) was associated with age (hazard ratio (HR) 1.04, 95% confidence interval (CI) 1.01–1.08, *p* = 0.0093), tumor grade (HR 3.64, 95% CI 1.63–9.63, *p* = 0.0010), genetic status (*p* < 0.0001), low gray-level run emphasis (LGRE, calculated from the gray-level run-length matrix) (HR 2.30 × 10^11^, 95% CI 737.11–4.23 × 10^19^, *p* = 0.0096), and correlation (calculated from the gray-level co-occurrence matrix) (HR 5.17, 95% CI 1.07–20.93, *p* = 0.041). The multivariate analyses revealed OS was independently associated with LGRE and correlation. The survival curves were also significantly different (both log-rank *p* < 0.05). Conclusion: Textural features obtained using preoperative MET positron emission tomography may compliment the semi-quantitative assessment for prognostication in glioma cases.

## 1. Introduction

Gliomas are the most common primary brain tumors and threaten the patient’s quality of life, cognitive function, and survival [1]. The prognosis for glioma remains dismal, despite advances in its diagnosis and the use of different therapeutic regimens for high-grade glioma. The 2016 World Health Organization (WHO) classification of gliomas identified three subtypes based on molecular and histological features: (1) diffuse isocitrate dehydrogenase (IDH) wild-type astrocytoma (IDH wild-type without 1p/19q-codeleted), (2) diffuse IDH mutant astrocytoma (IDH mutant without 1p/19q-codeleted), and (3) oligodendroglioma (IDH mutant with 1p/19q-codeleted) [2].

Positron emission tomography (PET) with amino acid analogs, especially ^11^C-methionine (MET), is well established in the diagnostic work-up of malignant brain tumors [3,4]. This is because MET can readily cross the blood–brain barrier through neutral amino acid transporters, which allows it to accumulate in active brain tumors. Furthermore, MET PET provides an advantage for imaging gliomas because the uptake of MET in normal brain tissue is lower than that of ^18^F-fluorodeoxyglucose (FDG) [5].

Texture analysis (TA) is a novel radiomics technique that is used to extract information regarding relationships among surrounding voxels, which is used to quantify their inhomogeneity. In addition, TA can evaluate intratumoral metabolic heterogeneity, which may vary in relation to the malignant potential of glioma. Recent TA-based studies of FDG PET findings have provided promising results for differentiating between benign and malignant tumors, in order to predict the treatment response and prognosis. However, to the best of our knowledge, only a few reports have described PET TA [6,7], especially regarding MET uptake, in patients with brain gliomas. Furthermore, there are few reports that have combined TA with genomic information (i.e., radiogenomics). The present study aimed to investigate whether TA applied to MET uptake has diagnostic and prognostic values in patients with glioma.

## 2. Methods

### 2.1. Study Subjects

This retrospective study included adult glioma patients who had undergone preoperative metabolic imaging at our hospital between February 2009 and April 2014. The inclusion criteria were as follows: (1) patients diagnosed with one or more brain lesions via magnetic resonance imaging, (2) patients who had undergone MET PET of the whole brain before surgery, (3) patients whose diagnosis was pathologically confirmed as glioma and who had complete molecular and histological data, (4) patients who were evaluated using the same PET/CT scanner as described below, and (5) patients with clear MET uptake that could be used to calculate the TA. The study’s retrospective protocol was approved by the ethics committee of Hokkaido University Hospital (IRB no. 015-0159) approved on 28 October 2015.

### 2.2. Image Acquisition and Reconstruction

All MET images were acquired using a Biograph 64 PET/CT scanner (Asahi-Siemens Medical Technologies, Ltd., Tokyo, Japan). The patients were instructed to fast for ≥3 h before the intravenous injection of MET (median: 370.5 MBq, interquartile range (IQR): 328.0–406.8 MBq). Whole-brain emission data were acquired for 10 min in the three-dimensional mode at 15–20 min after the MET injection. Attenuation-corrected images were reconstructed using the CT images and filtered back projection with a Hann filter (full-width at half-maximum of 4 mm) [3].

### 2.3. Image Analysis

An experienced nuclear medicine physician (K.K.), who was blinded to the pathological findings and patient outcomes, thoroughly reviewed the PET images and selected the MET-avid areas based on the coregistered fluid-attenuated inversion recovery MR images. The standardized uptake values (SUVs) were calculated as: [tissue radioactivity (Bq/mL)] × [body weight (g)] / [injected radioactivity (Bq)]. The tumor boundary was delineated using a threshold of 1.3× the reference SUVmean value, which was obtained by averaging the region of interest values (diameter: 10 mm) placed on the normal contralateral frontal lobe [3,8]. The tumor-to-normal ratio (TNR) was defined as the SUVmax divided by the reference value. Metabolic tumor volume (MTV) and total lesion methionine uptake (TLMU) were quantified as previously described [3], with MTV defined as the volume of the tumor boundary and TLMU defined as the product of the MTV and the SUVmean within the boundary. All image analyses were performed using the Metavol open source tool [3,9], and we resampled 64 discrete values from the lowest to the highest SUV values, which is a common method used in TA [10,11]. The PTexture package was used to calculate 36 texture features within the tumor boundary, as described by Orlhac et al. [10,12]. The complete source code for PTexture is available at www.github.com/metavol/ptexture.

### 2.4. MET-Guided Surgery and Postoperative Treatment

The MET-guided surgical procedure has been described previously [13]. Pathological data were obtained from biopsy tissue specimens or via cytoreduction performed after standard craniotomy under general anesthesia. Surgery was guided by intraoperative neuronavigation using a StealthStation TREON™ system (Medtronic, Louisville, CO, USA) for accurate resection at the tumor boundary, which was determined using multiple imaging modalities. For accurate biopsy targeting, silicone catheters were inserted at a target point within the region of the highest MET uptake according to the navigation findings from before the dural opening, in order to avoid inaccuracy caused by brain shift. Biopsy specimens were obtained via the inserted catheters during the tumor resection. The pathological diagnosis was performed by a neuropathologist based on the 2016 WHO classification [2]. We attempted to remove as much tumor tissue as possible in every patient, unless the tumor was confined to the deep brain structures (e.g., the thalamus) and/or involved eloquent brain areas (e.g., the perisylvian verbal area). All patients with pathologically confirmed grade III–IV glioma received additional chemoradiotherapy with temozolomide unless the additional therapy was contraindicated. The primary endpoint was overall survival (OS), which was calculated from surgery to all-cause death. A neurosurgeon (S.Y.) collected all data regarding pathological findings and patient outcomes.

### 2.5. Statistical Analysis

Continuous variables were expressed as median (interquartile range (IQR)) and categorical variables were expressed as number (percentage). All statistical analyses were performed using JMP software (version 14; SAS Institute, Cary, NC, USA). Differences were considered statistically significant at *p*-values of <0.05. Differences in semiquantitative parameters were analyzed using Wilcoxon’s signed rank test or the Kruskal–Wallis test. Spearman’s rank correlation test was used for inter-group comparisons. Heat map analysis with ascendant hierarchical clustering was performed to assess the relevance of each texture feature. After summarizing the clustering, the analyses used the features that had the highest cluster variation rate to explain the same group. The OS curves were compared using the Kaplan–Meier method and the long-rank test. Cox proportional hazard regression models were used to identify texture-based predictors of OS, with the multivariate models adjusted for age, WHO grade, and genetic status.

## 3. Results

### 3.1. Patient Characteristics and Outcomes

The present study included 42 patients (including 18 men) who had a median age of 56.0 years (IQR: 36.8–64.3 years). The WHO classification was used to histopathologically grade the gliomas, which were considered grade II for 7 patients (1 case of IDH mutant without 1p/19q-codeleted, 6 cases of IDH mutant with 1p/19q-codeleted), grade III for 17 patients (2 cases of IDH mutant without 1p/19q-codeleted, 7 cases of IDH mutant and 1p/19q-codeleted, 8 cases of IDH wild-type without 1p/19q-codeleted), and grade IV for 18 patients (18 cases of IDH wild-type without 1p/19q-codeleted) (Table 1). During the median follow-up of 18.8 months (IQR: 5.8–36.8 months), all-cause death was noted for 16 patients (38.1%).

### 3.2. MET PET Findings

The estimated MET parameters are presented in Table 2. Hierarchical clustering brought similar texture features close together in the heat map chart, with 6 features selected from the original 36 features: dissimilarity, low gray-level run emphasis (LGRE), high gray-level run emphasis (HGRE), gray-level non-uniformity for run (GLNUr), correlation (calculated from the gray-level co-occurrence matrix), and entropy (calculated from the gray-level co-occurrence matrix). There were no significant differences according to genetic status in the estimated values for TNR, CMV, TLMU, or the TA parameters.

### 3.3. Predictors of OS

**Figure 1:**
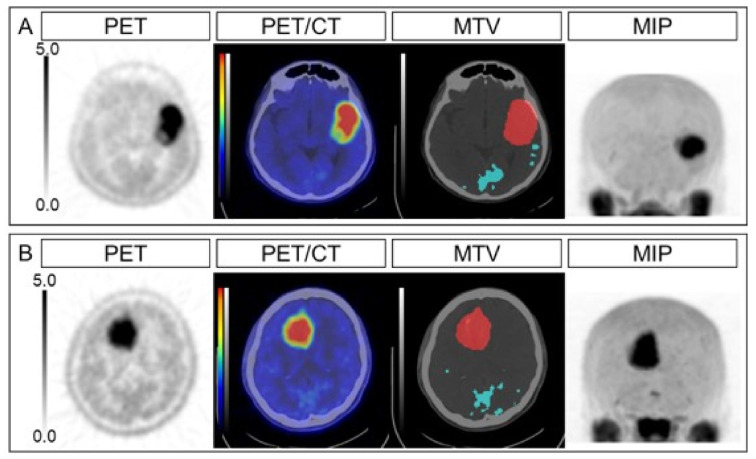
Representative cases. The PET, PET/CT, MTV, and MIP images are shown for an anaplastic astrocytoma case (**A**) and an anaplastic oligodendroglioma case (**B**). The SUVmax values were 7.34 and 6.87, the TNRs were 4.89 and 4.79, the MTVs were 52.7 mL and 56.3 mL, the TLMUs were 170.1 and 229.8, the LGREs were 0.118 and 0.054, and the correlations were 1.27 and 0.89, respectively, for cases (**A**) and (**B**). The overall survival intervals were 4.5 months (dead) and 31.6 months (surviving). PET, positron emission tomography; MTV, metabolic tumor volume; MIP, maximum intensity projection; SUVmax, maximum standardized uptake value; TNR, tumor-to-normal ratio; TLMU, total legion methionine uptake; LGRE, low gray-level run emphasis.

**Figure 2:**
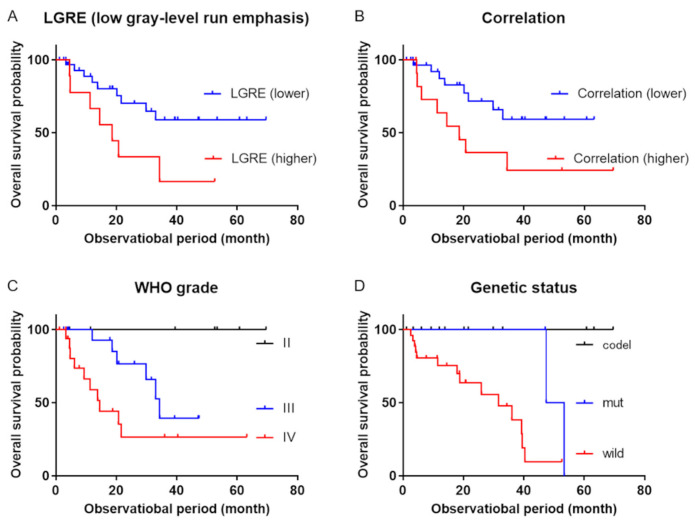
Kaplan–Meier survival curves. Kaplan–Meier survival curves revealed significant differences in overall survival between (**A**) patients with low gray-level run emphasis (LGRE) of <0.088 (blue line) and >0.088 (red line) (*p* = 0.022), (**B**) patients with correlation values of <1.074 (blue line) and >1.074 (red line), (**C**) patients with the different WHO grades (black line = grade II, blue line = grade III, red line = grade IV; *p* = 0.0066), and (**D**) patients with the different genetic statuses (black line = IDH mutant with 1p/19q-codeleted, blue line = IDH mutant without 1p/19q-codeleted, red line = IDH wild-type without 1p/19q-codeleted, *p* = 0.0004). WHO: World Health Organization; IDH: isocitrate dehydrogenase.

The univariate analyses revealed that OS was associated with age, tumor grade, genetic status, LGRE, and correlation, while OS was not significantly associated with sex, TNR, MTV, and TLMU (Table 3). The multivariable Cox proportional hazards models revealed that OS was independently associated with LGRE and correlation after adjusting for age (model 1), tumor grade (model 2), or genetic status (model 3) (all *p* < 0.05) (Table 4; Table 5). Representative cases are shown in Figure 1. The Kaplan–Meier curves revealed significantly better OS among patients with lower LGRE values (LGRE of <0.088; *n* = 33, *p* = 0.022) and lower correlation values (correlation of <1.074; *n* = 32, *p* = 0.03) (Figure 2).

## 4. Discussion

We investigated whether preoperative TA of MET PET data could predict the postoperative prognosis in patients with gliomas. The univariate and multivariate analyses demonstrated that LGRE and correlation, which are texture features derived from MET PET, were significant predictors of postoperative OS.

The present study used TA, which is a group of computational methods that can quantify inhomogeneity between adjacent voxels [14]. Since the early 1990s, the application of TA has been expanded using magnetic resonance imaging and CT for clinical morphological assessments [15,16]. When using PET, the FDG texture parameters of malignant tumors can help predict tumor progression and prognosis [17]. Among PET radiotracers, FDG is the most widely used and validated PET tracer, with this glucose analog being used to noninvasively assess the aggressiveness of various tumor types.

Glioma is the most common primary malignant brain tumor. However, high FDG accumulation in the normal surrounding brain tissue limits its utility during the imaging of brain tumors [18]. In this context, MET is a popular amino acid PET tracer that has relatively low uptake in normal brain tissue, and thus provides better detection of brain tumors (vs. FDG). Yu et al. have reported that the histogram features from MET PET can help detect O6-methylguanylmethyltransferase (MGMT) methylation, which can help predict the response to temozolomide-based chemotherapy [19]. Zhao et al. also reported that texture features from MET PET were able to help identify low-grade gliomas (IDH mutant and 1p/19q-codeleted), which have the most favorable outcomes [20]. Moreover, PET-based textural features obtained using amino acid tracers (e.g., ^18^F-fluoroethyl-L-tyrosine (FET) and 3′-deoxy-3′-^18^F-fluorothymidine (FLT)) can help characterize the glioma and predict survival outcomes [6,7].

The present study revealed that OS was significantly predicted by the correlation and LGRE values. Correlation values are calculated based on the gray-level co-occurrence matrix, using the Pearson’s correlation coefficients between adjacent voxels. Our data suggested that patients with higher correlation survived for less long. Higher correlation can be observed in tumors that show smaller value changes in voxel-by-voxel levels. Biologically, such images could represent infiltrative natures. On the other hand, The LGRE is a type of gray-level run-length matrix, which quantifies the size of homogeneous runs for each gray level. This parameter offers a size distribution of texture elements for a given direction in the image and is robust with respect to the delineation method, which allows it to describe the distribution of segments with relatively low accumulation within the boundary. High LGRE values are expected when there is a large number of segments with relatively low accumulation [21]. In the current study, patients with higher LGRE survived for less long. From a biological point of view, areas with low MET accumulation may be interpreted as necrotic foci, and thus high LGRE values might represent tumors with many small necrotic foci rather than few large ones. Our findings indicate that TA parameters based on MET uptake might reflect metabolic heterogeneity in gliomas, with correlation and LGRE potentially being useful as prognostic markers. A recent study by Papp et al. reported a machine-learning survival model for glioma (grade I to IV), combining MET PET radiomic features, histological information, and clinical information [22]. They reported that correlation was one of the most significant factors, ranking the third among texture features and the thirteenth among all the features. LGRE was not investigated in their study. A slight difference from the current study is that they found that zone-size non-uniformity, calculated from the gray-level zone-size matrix, was also a significant feature, which can be ascribed to different populations. In any event, we consider that texture analysis may contribute to predicting prognosis, although which features are most important remains to be clarified in larger studies.

### Limitations

This study has some methodological limitations. First, the retrospective design and small sample size are potential sources of bias. While requiring a sufficient sample size in order to conclude a valid research result, our finding will provide the basis for the development of future studies that may shape decision-making in this clinical setting. Second, while we only included patients who underwent the same PET/CT protocol at our hospital, the textural features of PET depend on the image acquisition conditions, with different acquisition modes and reconstruction parameters potentially influencing the results [23]. Third, oligodendroglial cell differentiation may influence MET uptake [4,24], although we were unable to evaluate this factor because of the limited number of patients. Further clinical studies are needed to address these issues and to confirm whether TA provides prognostic value in the clinical management of gliomas.

## 5. Conclusions

Textural variables obtained using MET PET may provide prognostic information that can complement semi-quantitative assessment in glioma cases.

## Figures and Tables

**Table 1:** Patient characteristics.

	Values
Age, years	56.0 (36.8–64.3)
Male sex	18 (43.5)
Karnofsky performance score	90 (70–92.5)
WHO grade, Ⅱ/Ⅲ/Ⅳ	7 (16.7)/17 (40.5)/18 (42.9)
Genetic status, wild/mut/codel	26 (61.9)/3 (7.1)/13 (31.0)
Surgical procedure, biopsy/resection	12 (28.6)/30 (71.4)

Values are reported as median (interquartile range) or *n* (%). WHO, World Health Organization; wild, isocitrate dehydrogenase (IDH) wild-type without 1p/19q-codeleted; mut, IDH mutant without 1p/19q-codeleted; codel, IDH mutant with 1p/19q-codeleted.

**Table 2:** The MET PET data.

	All (*n* = 42)	Wild (*n* = 26)	Mut (*n* = 3)	Codel (*n* = 13)
TNR	3.2 (2.4–3.9)	3.4 (2.7–4.1)	1.7 (1.5–3.6)	2.5 (1.8–4.1)
MTV (mL)	44.1 (10.9–92.6)	46.0 (18.8–92.6)	8.1 (6.0–190.3)	43.6 (4.9–81.8)
TLMU (mL)	121.2 (27.9–248.6)	280.3 (48.3–132.8)	14.2 (12.7–484.0)	89.8 (10.2–221.1)
**Texture features**				
Dissimilarity	6.4 (5.1–8.8)	6.2 (5.1–8.3)	12.2 (3.5–12.6)	6.5 (5.0–10.7)
LGRE	0.069 (0.054–0.084)	0.072 (0.059–0.087)	0.07 (0.05–0.10)	0.06 (0.05–0.08)
HGRE	531.6 (403.5–677.6)	535.7 (393.5–677.6)	544.2 (397.4–628.1)	520.2 (425.3–774.6)
GLNUr	104.9 (29.7–294.6)	111.5 (50.0–299.0)	16.5 (13.7–952.3)	119.9 (9.7–287.6)
Correlation	0.89 (0.75–1.10)	0.89 (0.77–1.11)	0.71 (0.51–1.22)	0.93 (0.70–1.08)
Entropy	6.7 (6.1–6.9)	6.7 (6.4–6.9)	6.06 (5.73–6.45)	6.43 (5.59–6.85)

Values are reported as median (interquartile range); wild, isocitrate dehydrogenase (IDH) wild-type without 1p/19q-codeleted; mut, IDH mutant without 1p/19q-codeleted; codel, IDH mutant with 1p/19q-codeleted; TNR, tumor-to-normal ratio; MTV, metabolic tumor volume; TLMU, total legion methionine uptake; LGRE, low gray-level run emphasis; HGRE, high gray-level run emphasis; GLNUr, gray-level non-uniformity for run.

**Table 3:** Univariate analyses of factors associated with all-cause death.

	Univariate Hazard Ratio (95% Confidence Interval)	*p*-Value
Age (per year)	1.04 (1.01–1.08)	0.0093
Male sex	2.71 (1.00–8.00)	0.051
WHO grade	3.64 (1.63–9.63)	0.0010
Genetic status	NA	<0.0001
TNR	1.34 (0.86–2.03)	0.19
MTV	1.01 (1.00–1.02)	0.086
TLMU	1.00 (1.00–1.01)	0.11
Dissimilarity	0.93 (0.77–1.09)	0.37
LGRE	2.30 × 10^11^ (737.11–4.23 × 10^19^)	0.0096
HGRE	1.00 (0.99–1.00)	0.11
GLNUr	1.00 (1.00–1.00)	0.27
Correlation	5.17 (1.07–20.93)	0.041
Entropy	0.77 (0.44–1.57)	0.45

WHO, World Health Organization; NA, not available because patients with IDH mutations and 1p/19q-codeleted had no events during the follow-up; TNR, tumor-to-normal ratio; MTV, metabolic tumor volume; TLMU, total legion methionine uptake; LGRE, low gray-level run emphasis; HGRE, high gray-level run emphasis; GLNUr, gray-level non-uniformity for run.

**Table 4:** Multivariate associations with all-cause death for LGRE.

	Model 1 χ^2^ = 12.8, *p* = 0.0017		Model 2 χ^2^ = 16.6, *p* = 0.0002		Model 3 χ^2^ = 27.3, *p* < 0.0001	
	Multivariate hazard ratio	*p*	Multivariate hazard ratio	*p*	Multivariate hazard ratio	*p*
Age	1.04 (1.01–1.08)	0.014	-	-	-	-
WHO grade	-	-	3.50 (1.56–9.29)	0.0017	-	-
Genetic status	-	-	–	-	NA	<0.0001
LGRE	2.60 × 10^10^ (147.20–4.11 × 10^18^)	0.014	1.06 × 10^11^ (144.02–5.38 × 10^19^)	0.016	9.07 × 10^11^ (508.99–1.28 × 10^21^)	0.012

WHO, World Health Organization; NA, not available because patients with IDH mutations and 1p/19q-codeleted had no events during the follow-up; LGRE, low gray-level run emphasis. Parentheses indicate the 95% confidence intervals.

**Table 5:** Multivariate associations with all-cause death for correlation.

	Model 1 χ^2^ = 11.2, *p* = 0.0037		Model 2 χ^2^ = 15.5, *p* = 0.0004		Model 3 χ^2^ = 27.6, *p* < 0.0001	
	Multivariate hazard ratio	*p*	Multivariate hazard ratio	*p*	Multivariate hazard ratio	*p*
Age	1.05 (1.01–1.09)	0.008	-	-	-	-
WHO grade	-	-	3.75 (1.68–9.92)	0.0008	-	-
Genetic status	-	-	-	-	NA	<0.0001
Correlation	5.31 (1.13–21.82)	0.035	5.48 (1.21–22.6)	0.029	7.03 (1.63–29.2)	0.011

WHO, World Health Organization; NA, not available because patients with IDH mutations and 1p/19q-codeleted had no events during the follow-up. Parentheses indicate the 95% confidence intervals.

## Data Availability

All relevant data are included in the manuscript and related files.

## References

[1] Omuro A., DeAngelis L.M. (2013). Glioblastoma and other malignant gliomas: A clinical review. JAMA.

[2] Louis D.N., Perry A., Reifenberger G., Von Deimling A., Figarella-Branger D., Cavenee W.K., Ohgaki H., Wiestler O.D., Kleihues P., Ellison D.W. (2016). The 2016 World Health Organization Classification of Tumors of the Central Nervous System: A summary. Acta Neuropathol..

[3] Kobayashi K., Hirata K., Yamaguchi S., Manabe O., Terasaka S., Kobayashi H., Shiga T., Hattori N., Tanaka S., Kuge Y. (2015). Prognostic value of volume-based measurements on 11C-methionine PET in glioma patients. Eur. J. Nucl. Med. Mol. Imaging.

[4] Manabe O., Hattori N., Yamaguchi S., Hirata K., Kobayashi K., Terasaka S., Kobayashi H., Motegi H., Shiga T., Magota K. (2015). Oligodendroglial component complicates the prediction of tumour grading with metabolic imaging. Eur. J. Nucl. Med. Mol. Imaging.

[5] Singhal T., Narayanan T.K., Jacobs M.P., Bal C., Mantil J.C. (2012). 11C-Methionine PET for Grading and Prognostication in Gliomas: A Comparison Study with 18F-FDG PET and Contrast Enhancement on MRI. J. Nucl. Med..

[6] Pyka T., Gempt J., Hiob D., Ringel F., Schlegel J., Bette S., Wester H.-J., Meyer B., Förster S. (2015). Textural analysis of pre-therapeutic [18F]-FET-PET and its correlation with tumor grade and patient survival in high-grade gliomas. Eur. J. Nucl. Med. Mol. Imaging.

[7] Mitamura K., Kudomi N., Maeda Y., Norikane T., Miyake K., Nishiyama Y., Yamamoto Y. (2016). Intratumoral heterogeneity of 18F-FLT uptake predicts proliferation and survival in patients with newly diagnosed gliomas. Ann. Nucl. Med..

[8] Galldiks N., Ullrich R.T., Schroeter M., Fink G.R., Kracht L.W. (2009). Volumetry of [11C]-methionine PET uptake and MRI contrast enhancement in patients with recurrent glioblastoma multiforme. Eur. J. Nucl. Med. Mol. Imaging.

[9] Hirata K., Kobayashi K., Wong K.-P., Manabe O., Surmak A., Tamaki N., Huang S.-C. (2014). A Semi-Automated Technique Determining the Liver Standardized Uptake Value Reference for Tumor Delineation in FDG PET-CT. PLoS ONE.

[10] Manabe O., Ohira H., Hirata K., Hayashi S., Naya M., Tsujino I., Aikawa T., Koyanagawa K., Oyama-Manabe N., Tomiyama Y. (2018). Use of 18F-FDG PET/CT texture analysis to diagnose cardiac sarcoidosis. Eur. J. Nucl. Med. Mol. Imaging.

[11] Kroenke M., Hirata K., Gafita A., Watanabe S., Okamoto S., Magota K., Shiga T., Kuge Y., Tamaki N. (2019). Voxel based comparison and texture analysis of 18F-FDG and 18F-FMISO PET of patients with head-and-neck cancer. PLoS ONE.

[12] Orlhac F., Thézé B., Soussan M., Boisgard R., Buvat I. (2016). Multiscale Texture Analysis: From 18F-FDG PET Images to Histologic Images. J. Nucl. Med..

[13] Yamaguchi S., Kobayashi H., Hirata K., Shiga T., Tanaka S., Murata J., Terasaka S. (2010). Detection of histological anaplasia in gliomas with oligodendroglial components using positron emission tomography with 18F-FDG and 11C-methionine: Report of two cases. J. Neuro-Oncology.

[14] Orlhac F., Soussan M., Maisonobe J.A., Garcia C.A., Vanderlinden B., Buvat I. (2014). Tumor texture analysis in 18F-FDG PET: Relationships between texture parameters, histogram indices, standardized uptake values, metabolic volumes, and total lesion glycolysis. J. Nucl. Med..

[15] Schad L.R., Blüml S., Zuna I. (1993). MR tissue characterization of intracranial tumors by means of texture analysis. Magn. Reson. Imaging.

[16] Mir A.H., Hanmandlu M., Tandon S.N. (1995). Texture analysis of CT-images for early detection of liver malignancy. Biomed. Sci. Instrum..

[17] Nakajo M., Jinguji M., Shinaji T., Aoki M., Tani A., Nakabeppu Y., Nakajo M., Sato M., Yoshiura T. (2018). A Pilot Study of Texture Analysis of Primary Tumor [18F]FDG Uptake to Predict Recurrence in Surgically Treated Patients with Non-small Cell Lung Cancer. Mol. Imaging Biol..

[18] Glaudemans A.W.J.M., Enting R.H., Heesters M.A.A.M., Dierckx R.A.J.O., Van Rheenen R.W.J., Walenkamp A.M.E., Slart R.H.J.A. (2013). Value of 11C-methionine PET in imaging brain tumours and metastases. Eur. J. Nucl. Med. Mol. Imaging.

[19] Yu P., Ning J., Xu B., Liu J., Dang H., Lin M., Feng X., Grimm R., Tian J. (2019). Histogram analysis of 11C-methionine integrated PET/MRI may facilitate to determine the O6-methylguanylmethyltransferase methylation status in gliomas. Nucl. Med. Commun..

[20] Zhao K., Yu P., Xue Z., Liu J., Yao A., Zhao Y., Yang F., Tian J., Xu B. (2020). (11)C-Methionine Integrated PET/MRI-Based Texture Analysis Features May Have a Potential Ability to Distinguish Oligodendroglioma (IDH-Mutant and 1p/19q-Codeleted) From Varied Gliomas. Acad Radiol..

[21] Soussan M., Orlhac F., Boubaya M., Zelek L., Ziol M., Eder V., Buvat I. (2014). Relationship between Tumor Heterogeneity Measured on FDG-PET/CT and Pathological Prognostic Factors in Invasive Breast Cancer. PLoS ONE.

[22] Papp L., Poetsch N., Grahovac M., Schmidbauer V., Woehrer A., Preusser M., Mitterhauser M., Kiesel B., Wadsak W., Beyer T. (2017). Glioma Survival Prediction with Combined Analysis of In Vivo11C-MET PET Features, Ex Vivo Features, and Patient Features by Supervised Machine Learning. J. Nucl. Med..

[23] Hatt M., Tixier F., Pierce L., Kinahan P.E., Le Rest C.C., Visvikis D. (2017). Characterization of PET/CT images using texture analysis: The past, the presen any future?. Eur. J. Nucl. Med. Mol. Imaging.

[24] Yano H., Ohe N., Nakayama N., Nomura Y., Miwa K., Shinoda J., Iwama T. (2015). Dynamic study of methionine positron emission to-mography in patients with glioblastoma with oligodendroglial components. Brain Tumor Pathol..

